# Cooperative Binding of KaiB to the KaiC Hexamer Ensures Accurate Circadian Clock Oscillation in Cyanobacteria

**DOI:** 10.3390/ijms20184550

**Published:** 2019-09-13

**Authors:** Reiko Murakami, Yasuhiro Yunoki, Kentaro Ishii, Kazuki Terauchi, Susumu Uchiyama, Hirokazu Yagi, Koichi Kato

**Affiliations:** 1 Graduate School of Pharmaceutical Sciences, Nagoya City University, 3-1 Tanabe-dori, Mizuho-ku, Nagoya 467-8603, Japan; taniyama@fujita-hu.ac.jp (R.M.); c182803@ed.nagoya-cu.ac.jp (Y.Y.); kishii@bio.eng.osaka-u.ac.jp (K.I.); hyagi@phar.nagoya-cu.ac.jp (H.Y.); 2 Institute for Molecular Science, National Institutes of Natural Sciences, 5-1 Higashiyama, Myodaiji-cho, Okazaki 444-8787, Japan; 3 Department of Creative Research, Exploratory Research Center on Life and Living Systems (ExCELLS), National Institutes of Natural Sciences, 5-1 Higashiyama, Myodaiji-cho, Okazaki 444-8787, Japan; suchi@bio.eng.osaka-u.ac.jp; 4 Graduate School of Life Sciences, Ritsumeikan University, 1-1-1 Noji-higashi, Kusatsu, Shiga 525-8577, Japan; terauchi@fc.ritsumei.ac.jp; 5 Department of Biotechnology, Graduate School of Engineering, Osaka University, 2-1 Yamadaoka, Suita, Osaka 565-0871, Japan

**Keywords:** cooperative interaction, circadian rhythm, clock protein, native mass spectrometry, cooperativity

## Abstract

The central oscillator generating cyanobacterial circadian rhythms comprises KaiA, KaiB, and KaiC proteins. Their interactions cause KaiC phosphorylation and dephosphorylation cycles over approximately 24 h. KaiB interacts with phosphorylated KaiC in competition with SasA, an output protein harboring a KaiB-homologous domain. Structural data have identified KaiB–KaiC interaction sites; however, KaiB mutations distal from the binding surfaces can impair KaiB–KaiC interaction and the circadian rhythm. Reportedly, KaiB and KaiC exclusively form a complex in a 6:6 stoichiometry, indicating that KaiB–KaiC hexamer binding shows strong positive cooperativity. Here, mutational analysis was used to investigate the functional significance of this cooperative interaction. Results demonstrate that electrostatic complementarity between KaiB protomers promotes their cooperative assembly, which is indispensable for accurate rhythm generation. SasA does not exhibit such electrostatic complementarity and noncooperatively binds to KaiC. Thus, the findings explain KaiB distal mutation effects, providing mechanistic insights into clock protein interplay.

## 1. Introduction

Circadian rhythms enable organisms, including mammals and plants, to coordinate their biochemistry and physiology with daily environmental alterations that are caused by Earth’s light and dark cycles. Cyanobacteria are photoautotrophic organisms that use circadian rhythms to regulate their photosynthetic activity. A similar regulatory mechanism has been observed in some eukaryotic algae and plants; accordingly, cyanobacteria are considered to be one of the most useful model organisms to study circadian biology [1]. Cyanobacteria utilize a central oscillator to maintain their circadian rhythms. It comprises three proteins—KaiA, KaiB, and KaiC [2]—which interact in the presence of adenosine 5′-triphosphate (ATP), resulting in periodic KaiC phosphorylation and dephosphorylation cycles over 24 h. The KaiABC circadian clock has been successfully reconstituted in vitro without daylight oscillation, suggesting that the clock functions autonomously, irrespective of transcriptional and translational feedback systems [3]. 

**Figure 1:**
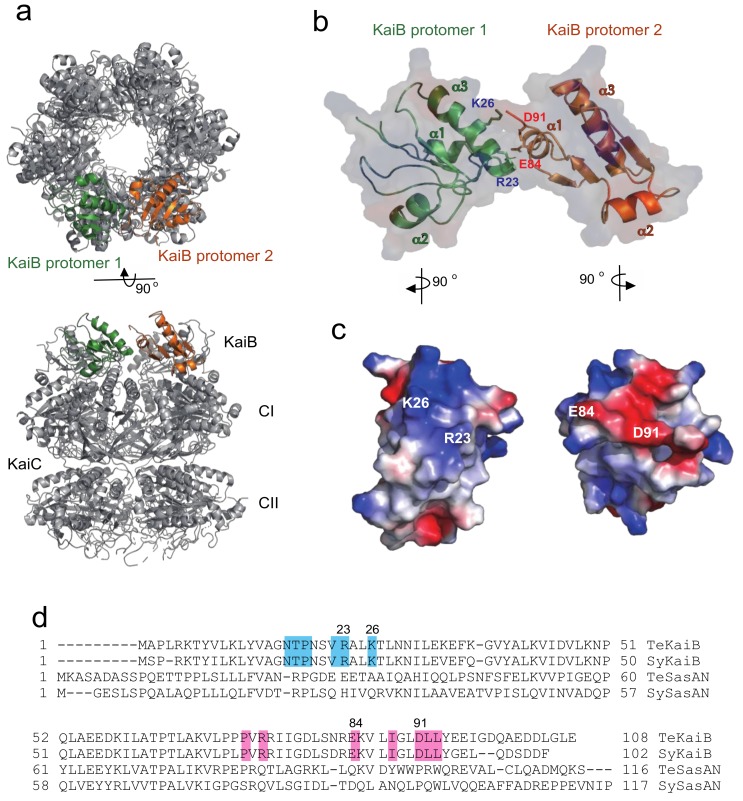
KaiB–KaiB interface in the KaiB–KaiC complex. (**a**) Overall structure of the KaiB–KaiC complex. This structural model is based on the amino acid sequence of wild-type TeKaiB and the crystal structure of the complex in which TeKaiB has three mutation sites (PDB code: 5JWQ). The KaiC hexamer forms a double-ring structure composed of CI and CII rings and KaiB forms a hexameric ring on the CI ring. The two KaiB protomers are colored green and orange. (**b**,**c**) The KaiB–KaiB lateral interaction and surface electrostatic complementarity between two KaiB protomers. (**d**) Sequence alignment of KaiB and SasA originating from *Synechococcus elongatus* PCC 7942 and *Thermosynechococcus elongatus*. Sequences were aligned using the Dali server. The residues involved in the KaiB–KaiB lateral interactions are highlighted in cyan (protomer 1) and pink (protomer 2).

KaiC forms a homohexamer with a double-ring structure composed of the CI and CII rings (Figure 1a) [4,5]. Two specific residues, Ser431 and Thr432 (denoted as S and T, respectively), that are positioned in the CII ring are periodically phosphorylated and dephosphorylated over 24 h as follows: KaiC-S/T → KaiC-S/pT → KaiC-pS/pT → KaiC-pS/T → KaiC-S/T, where “p” represents the phosphorylated residue [6]. KaiA stimulates KaiC phosphorylation through its interaction with the C-terminal region of the CII ring [5,7,8], whereas KaiB preferentially binds to the CI ring of phosphorylated KaiC (p-KaiC), which accelerates dephosphorylation [6,9,10,11]. 

SasA is a circadian clock-output protein with an N-terminal domain that is homologous to KaiB and a C-terminal EnvZ-like histidine kinase domain [12,13]. It interacts with the CI ring of the phosphorylated KaiC hexamer using its N-terminal domain [12], which triggers the autophosphorylation of SasA at its catalytic histidine residue (e.g., His160 in *Thermosynechococcus elongatus* SasA). The phosphorylated SasA is released from KaiC, and it subsequently interacts with and transfers the phosphate group to its cognate response regulator, RpaA [14]. The phosphorylated RpaA then regulates the transcription of its target genes that are associated with circadian rhythms. KaiB and SasA have a common interaction surface on the CI ring of the phosphorylated KaiC and therefore compete with each other to bind to KaiC [15,16,17].

Previous structural studies regarding the KaiB–KaiC interaction using crystallography, electron microscopy, solution scattering, and native mass spectrometry (MS) have revealed that the KaiC hexamer can accommodate a maximum of six KaiB protomers (Figure 1a). The crystallographic data revealed that the KaiB–KaiC interaction sites are mainly composed of a KaiC B-loop and KaiB α2 helix. Intriguingly, mutations in KaiB that are distal from these binding surfaces, e.g., R22A and D90G in *Synechococcus elongatus* KaiB, can impair its interaction with the KaiC hexamer and the consequent circadian rhythm [18,19]. These mutations are located at the lateral KaiB–KaiB interaction surfaces in the KaiB–KaiC complex [20]. In a previous study, native MS titration analysis revealed that KaiB and KaiC form a complex exclusively in a 6:6 stoichiometry, indicating that KaiB and the KaiC hexamer bind with strong positive cooperativity [21]. However, the biological relevance of this cooperative interaction remains to be elucidated.

Here, the molecular mechanisms and functional significance of the cooperative binding of KaiB to the KaiC hexamer are addressed by mutational analysis using native MS. In addition, the KaiC-binding property of SasA compared with that of KaiB is characterized. Finally, based on the findings, the importance of the cooperative binding of KaiB to the KaiC hexamer for regulating circadian rhythms is discussed.

## 2. Results and Discussion

### 2.1. Complex Formation of KaiC with KaiB Mutants

First, the identification of structural factors contributing to the mechanism of cooperative binding of KaiB to the KaiC hexamer was attempted. The crystal structure of the KaiB–KaiC complex (PDB code: 5JWQ) revealed that six KaiB molecules are arranged into a ring structure on the KaiC CI ring, with each KaiB protomer in contact with its neighboring KaiB protomers (Figure 1a) [20]. In the lateral KaiB–KaiB interface, interactions between each KaiB molecule in the ring involve the binding of positively charged residues (Arg23 and Lys26) of the α3 helix of one KaiB protomer with negatively charged residues (Glu84 and Asp91) of the α1 helix of its clockwise neighbor (Figure 1b,c) [20]. The amino acid residues involved in interaction surfaces are conserved among cyanobacteria (Figure 1d and Figure S1). The importance of electrostatic complementarity in the KaiB–KaiC interaction was examined by mutational experiments in conjunction with native MS analysis. For this purpose, the basic amino acid residues, Arg23 and Lys26, were both replaced with aspartate in an N-terminal truncated mutant of *T. elongatus* KaiB (termed TeKaiB_10–108_), which has been reported to be monomeric and a higher-affinity binder to its cognate KaiC than the wild-type protein [16,22]. Therefore, we deleted the N-terminal segment of KaiB to facilitate native MS detection of the KaiB–KaiC complex. The mutant thus constructed was designated as TeKaiB_10–108/DD_. We confirmed that the DD mutation affected neither the secondary structure nor tetramer formation of TeKaiB in the absence of KaiC (Figure S2). Further, we used a *T. elongatus* KaiC mutant with its Ser431 and Thr432 substituted by aspartate residues (TeKaiC_DD_) because this KaiC mutant mimics the fully phosphorylated form and exhibits a higher binding affinity to TeKaiB and its monomeric mutants than those that mimic the nonphosphorylated form [22].

**Figure 2:**
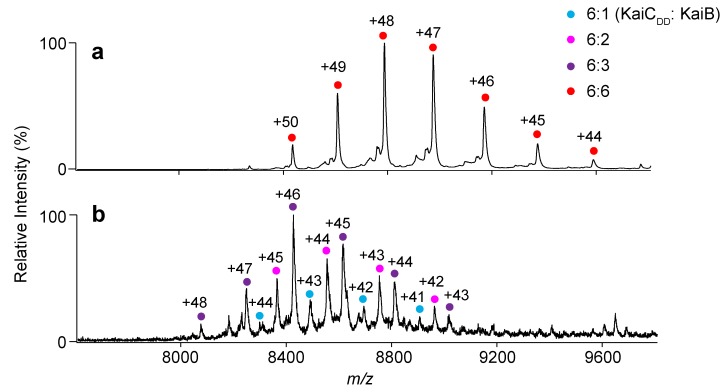
Native MS analysis of KaiB–KaiC complex formation. Mass spectra of mixtures of TeKaiC_DD_ and (**a**) TeKaiB_10–108_ or (**b**) TeKaiB_10–108/DD_ at a 1:3.5 molar ratio (TeKaiC_DD_ to TeKaiB). The cyan, magenta, purple, and red circles show the ion series of 6:1, 6:2, 6:3, and 6:6 complexes of TeKaiC_DD_ and TeKaiB mutants, respectively.

The MS titration data confirmed that TeKaiB_10–108_ and TeKaiC_DD_ formed a complex exclusively in a 6:6 stoichiometry (Figure 2a and Figure S3a). This indicates that six TeKaiB protomers bind to the TeKaiC hexamer with strong positive cooperativity, which is consistent with previous observations obtained using synechococcal Kai (SyKai) proteins [21,23]. In contrast, TeKaiB_10–108/DD_ formed complexes with TeKaiC_DD_ in varying stoichiometries from 1:6 to 3:6 (Figure 2b and Figure S3b). However, in comparison with TeKaiB_10–108_, which has a native lateral interface, TeKaiB_10–108/DD_ showed virtually no difference in its binding affinity to monomeric KaiC (Figure S4). These results indicated that the electrostatic complementarity at the lateral KaiB–KaiB interface promotes the cooperative assembly of KaiB protomers on the KaiC hexamer.

### 2.2. Complex Formation of KaiC with SasA

**Figure 3:**
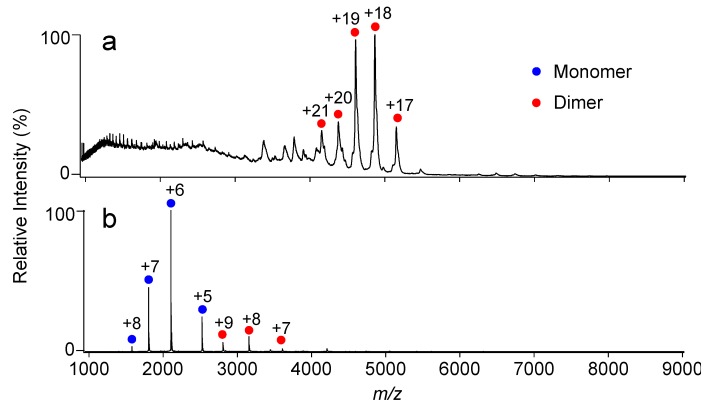
Native MS analysis of SasA and its mutant. Mass spectra of (**a**) TeSasA and (**b**) TeSasA_N_. The blue and red circles show the ion series of the SasA monomer and dimer, respectively.

**Figure 4:**
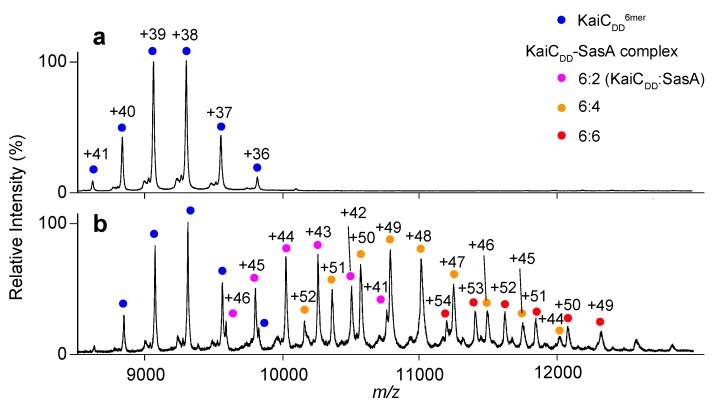
Native MS analysis of TeKaiC_DD_–TeSasA complex formation. Mass spectra of mixtures of TeKaiC_DD_ and TeSasA at (**a**) 1:0 and (**b**) 1:1 molar ratios (TeKaiC_DD_ to TeSasA). The blue, magenta, orange, and red circles show the ion series of the TeKaiC_DD_ homohexamer and 6:2, 6:4, and 6:6 complexes of TeKaiC_DD_ and TeSasA, respectively.

The N-terminal domain of SasA adopts a thioredoxin fold that is homologous to the KaiC-bound form of KaiB (Figure S5). This suggests that KaiB and SasA share a KaiC binding site [15,24]. Intriguingly, most amino acid residues involved in the lateral KaiB–KaiB interactions, including the complementary charged residues, are not conserved in SasA (Figure 1d). This prompted the investigation of whether SasA can bind to KaiC hexamers in a cooperative manner. The KaiC mutant TeKaiC_DD_ has the highest affinity for SasA among mutants mimicking the phosphorylated state [16,17]. Therefore, TeKaiC_DD_ was used for analyzing its interaction with TeSasA. Native MS analysis indicated TeSasA formed a dimer (Figure 3a), which is in contradiction to the previous report describing that the full-length SasA forms a trimer based on size exclusion chromatography and sedimentation equilibrium analyses [17]. This discrepancy might be attributed to the elongated, non-globular structure of full-length SasA and co-existence of higher oligomers that were detected in the chromatographic analysis in the previous solution condition, which hinder interpretation of those hydrodynamic data. Native MS-based titration of TeKaiC_DD_ with TeSasA detected TeSasA/TeKaiC_DD_ complexes in 2:6, 4:6, and 6:6 stoichiometries, suggesting that TeSasA interacts with KaiC as dimer (Figure 4 and Figure S6a). To eliminate potential ambiguities in data interpretation caused by the dimerization property of TeSasA, we used the N-terminal domain of SasA (TeSasA_N_), which was confirmed to be monomeric (Figure 3b), consistent with the previous report [17].

**Figure 5:**
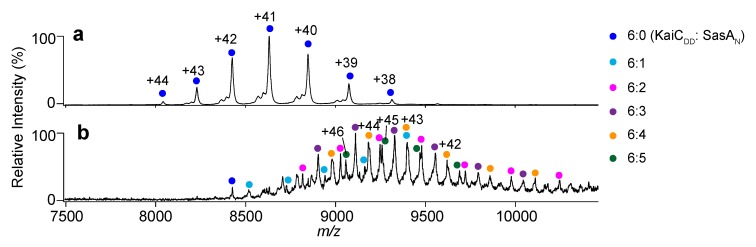
Native MS analysis of TeKaiC_DD_–TeSasA_N_ complex formation. Mass spectra of mixtures of TeKaiC_DD_ and TeSasA_N_ at (**a**) 1:0 and (**b**) 1:3.5 molar ratios (TeKaiC_DD_ to TeSasA_N_). The blue, cyan, magenta, purple, orange, and green circles show the ion series of the TeKaiC_DD_ homohexamer and 6:1, 6:2, 6:3, 6:4, and 6:5 complexes of TeKaiC_DD_ and TeSasA_N_, respectively.

Titration of TeKaiC_DD_ with TeSasA_N_ gave rise to their complexes in varying stoichiometries from 6:1 to 6:5 (Figure 5 and Figure S6b). This indicated that, in marked contrast to the KaiB–KaiC interaction, the SasA N-terminal domain exhibited no obvious binding cooperativity to the KaiC hexamer. The cooperative binding of KaiB to the KaiC hexamer may cause a synergistic release of SasA from the oscillator complex for strong downstream signaling to regulate the circadian rhythm.

### 2.3. Effects of KaiB Mutantation on Circadian Oscillations

To address the functional relevance of the cooperative KaiB–KaiC interaction, an in vitro clock system of SyKai proteins [25] of wild-type and mutant KaiB was used to examine if the disruption of charge complementarity at the lateral KaiB–KaiB interface showed any effects on circadian oscillation. We confirmed the DD mutation of SyKaiB impaired its cooperative interaction with KaiC_DT_ (Figure S7).

**Figure 6:**
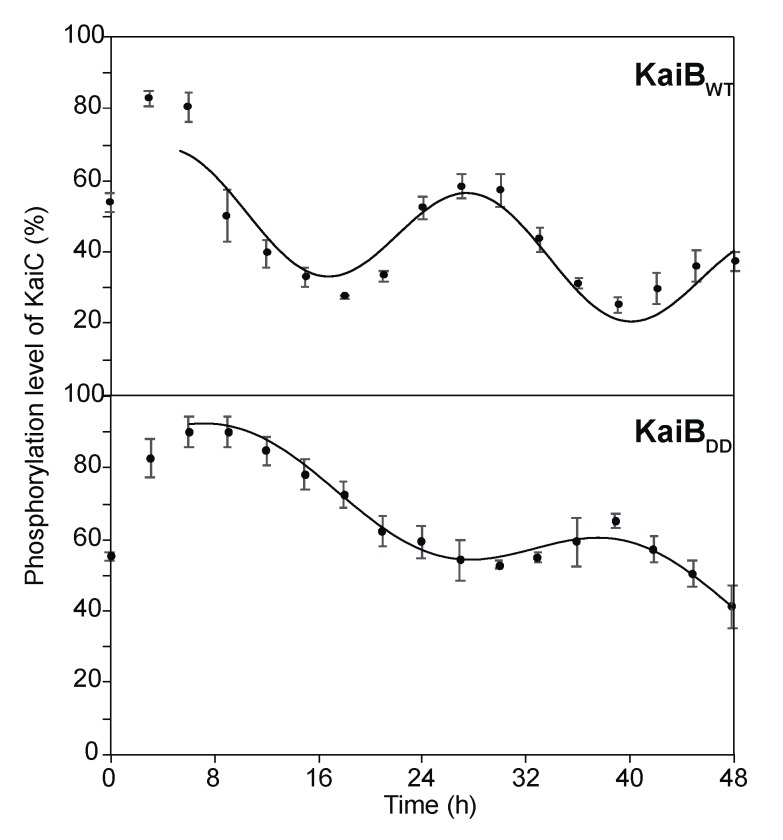
Circadian oscillations in vitro. Phosphorylated KaiC levels in the presence of wild-type SyKaiB and its mutant SyKaiB_DD_. Plots show the percentage ratios of phosphorylated KaiC to total KaiC in the presence of KaiB_WT_ and KaiB_DD_. The relative amounts of unphosphorylated and phosphorylated KaiC were estimated using densitometry. The data represent the means ± SD from three independent experiments. The simulated time course showing circadian oscillations in the phosphorylated KaiC level were generated using an integrated rhythm-analyzing program [27].

As shown in Figure 6 and Figure S8, the SyKaiB_DD_ mutant perturbed the periodic oscillation of the phosphorylated KaiC level, which resulted in prolonged time periods of the circadian rhythm, i.e., 30.4 h, in comparison with the wild type (23.2 h). Furthermore, the mutation caused considerable reduction in the amplitude of the circadian oscillation. These results indicate that the electrostatic complementarity at the KaiB–KaiB interaction surface is a determining factor for an accurate circadian clock oscillation. Previous studies have reported that a KaiB R22A mutation decreased its affinity for the KaiC hexamer and a KaiB D90G mutation severely impaired the *S. elongatus* PCC 7942 circadian rhythm [19]. Because these mutations can compromise the charge complementarity between the two KaiB protomers, the observed effects can be explained by loss or attenuation of the positive cooperativity of KaiB–KaiC hexamer binding. 

The KaiB hexameric ring formed on the KaiC hexamer can bind KaiA molecules [20,26], resulting in a rapid depletion of free KaiA molecules in the clock system. Therefore, cooperative binding of the KaiB–KaiC hexamer may facilitate a robust clock oscillation by enabling the efficient sequestration of KaiA molecules. 

The findings suggest that the cooperative binding of the KaiB protomers to the KaiC hexamer enables sharp phase switching of the circadian oscillator complex through the synergistic release of the SasA clock-output protein and the coincidental sequestration of the KaiA phosphorylation enhancer. These mechanisms contribute to the synchronous nature of the cyanobacterial circadian system.

## 3. Materials and Methods 

### 3.1. Protein Expression and Purification

KaiA, KaiB, KaiC, and SasA from *Synechococcus elongatus* PCC 7942 and *T. elongatus* were expressed in *Escherichia coli.* SyKaiA and SyKaiC were expressed and purified as Strep-tagged recombinant proteins [21,28]. TeKaiA, TeKaiB, TeKaiC, TeSasA, and SyKaiB were expressed as glutathione S-transferase (GST)-tagged recombinant proteins [4] and purified after the cleavage of the GST-tag, as described previously [16,21]. The mutated proteins, TeKaiB_10–108_, TeKaiB_10–108/DD_, SyKaiB_DD_, SyKaiC_DT_, TeKaiC_DD_, and SasA_N_ were expressed and purified by the same method used for their wild-type counterparts [16,22,29,30]. For the KaiB mutant, the basic amino acid residues, Arg23 and Lys26, in TeKaiB_10–108_ and the corresponding Arg22 and Lys25 in SyKaiB were substituted with aspartate residues.

### 3.2. Size Exclusion Chormatography

Size exclusion chromatography analysis of TeKaiB or TeKaiB_DD_ was performed with a Superdex 200 increase column (GE Healthcare Japan, Tokyo, Japan) equilibrated with 20 mM Tris-HCl (pH 8.0) containing 150 mM NaCl, 5 mM MgCl_2_, 0.5 mM EDTA and 1 mM DTT at 0.75 min/mL flow rate. The elution profiles of proteins were monitored by absorbance at 280 nm.

### 3.3. Circular Dichroism Spectra

TeKaiB_10–108_ or TeKaiB_10–108/DD_ (0.2 mg/mL) was dissolved in 20 mM phosphate buffer (pH 7.8) containing 150 mM NaCl. Measurements of circular dichroism spectra were performed in a 1-mm quartz cuvette at a room temperature using a spectropolarimeter (J-725, JASCO, Tokyo, Janan). After subtraction of the spectrum of the buffer alone, data were represented as mean residue ellipticities.

### 3.4. Native MS Analysis

The purified TeKaiC_DD_ (20 µM) was titrated with either KaiB proteins (TeKaiB_10–108_ and TeKaiB_10–108/DD_) or SasA proteins (TeSasA_WT_ and TeSasA_N_). The mixed protein solutions were incubated at 37 °C for 6 h, and were subsequently buffer-exchanged into 150 mM ammonium acetate, pH 6.8, using a Bio-Spin 6 column (Bio-Rad, Hercules, CA, USA) according to the manufacturer’s instructions. In analyses of the Kai proteins from *Synechococcus elongatus* PCC 7942, the purified SyKaiC_DT_ (20 µM) was mixed with SyKaiB or SyKaiB_DD_ (30 µM), followed by incubation at 30 °C for 6 h. Approximately 2–5 µL of the buffer-exchanged protein solutions were immediately analyzed by nanoflow electrospray ionization MS using gold-coated glass capillaries prepared inhouse. Spectra were recorded on a SYNAPT G2-Si HDMS (Waters, Wilmslow, UK) in the positive ionization mode at 1.33 kV with a 150 V sampling cone voltage and source offset voltage, 0-V trap and transfer collision energy, and 5-mL/min trap gas flow. The spectra were calibrated using 1 mg/mL cesium iodide and analyzed using MassLynx software (Waters). Spectral measurements were performed at least in triplicate.

### 3.5. Measurement of Time-dependent Phosphorylated KaiC Levels

Reaction mixtures of SyKaiA (1.5 µM) and SyKaiC (3.4 µM) were incubated in the presence of 4.2 µM SyKaiB_WT_, or SyKaiB_DD_ at 30 °C, and 3 μL aliquots of the reaction mixtures were removed to stop the reaction at specific time intervals, as described previously [28]. The aliquots were analyzed using sodium dodecyl sulfate polyacrylamide gel electrophoresis (SDS-PAGE) and Coomassie Brilliant Blue staining. The intensities of the bands were measured by densitometry using ImageJ 1.41 software (National Institutes of Health). The upper bands corresponded to phosphorylated KaiC, and the lower band corresponded to nonphosphorylated KaiC [25]. The relative proportions of phosphorylated KaiC to total KaiC (phosphorylated KaiC level) from the band intensities were calculated and the circadian oscillations associated with each phosphorylated KaiC level were analyzed using the rhythm-analyzing program [27].

## Supplementary Materials

The Supplementary materials can be found at https://www.mdpi.com/1422-0067/20/18/4550/s1.

## Abbreviations

ATP	adenosine 5′-triphosphate
p-KaiC	phosphorylated KaiC
MS	mass spectrometry
Te	*Thermosynechococcus elongatus* (strain BP-I)
Sy	*Synechococcus elongatus* (strain PCC 7942)
SDS-PAGE	sodium dodecyl sulfate polyacrylamide gel electrophoresis
