# Supplementary material for: Cooperative Binding of KaiB to the KaiC Hexamer Ensures Accurate Circadian Clock Oscillation in Cyanobacteria
Source: Int J Mol Sci. 2019 Sep 13;20(18):4550. doi: 10.3390/ijms20184550 (PMC6769508; doi:10.3390/ijms20184550)
Supplement: Supplementary file 1 [file ijms-20-04550-s001.pdf]

Supplemental Information: Cooperative Binding of KaiB to the KaiC Hexamer Ensures Accurate Circadian Clock Oscillation in Cyanobacteria

Reiko Murakami [1,†,‡], Yasuhiro Yunoki [1,2,‡], Kentaro Ishii [1,2,3], Kazuki Terauchi [4], Susumu Uchiyama [3,5], Hirokazu Yagi [1] and Koichi Kato [1,2,3,*]

[1] Graduate School of Pharmaceutical Sciences, Nagoya City University, 3-1 Tanabe-dori, Mizuho-ku, Nagoya 467-8603, Japan; taniyama@fujita-hu.ac.jp (R.M.); c182803@ed.nagoya-cu.ac.jp (Y.Y.); kishii@bio.eng.osaka-u.ac.jp (K.I.); hyagi@phar.nagoya-cu.ac.jp (H.Y.)

[2] Institute for Molecular Science, National Institutes of Natural Sciences, 5-1 Higashiyama, Myodaiji-cho, Okazaki 444-8787, Japan

[3] Department of Creative Research, Exploratory Research Center on Life and Living Systems (ExCELLS), National Institutes of Natural Sciences, 5-1 Higashiyama, Myodaiji-cho, Okazaki 444-8787, Japan; suchi@bio.eng.osaka-u.ac.jp

[4] Graduate School of Life Sciences, Ritsumeikan University, 1-1-1 Noji-higashi, Kusatsu, Shiga 525-8577, Japan; terauchi@fc.ritsumei.ac.jp

[5] Department of Biotechnology, Graduate School of Engineering, Osaka University, 2-1 Yamadaoka, Suita, Osaka 565-0871, Japan

* Correspondence: kkato@excells.orion.ac.jp; Tel.: +81-564-59-5225; Fax: +81-564-59-5224

† Present address: Research Promotion and support headquarters, Fujita Health University Graduate School of Health Sciences, Toyoake, Aichi 470-1192, Japan

‡ These two authors contributed equally to this work.

Supplemental Figures 1, 2, 3, 4, 5, 6, 7, and 8

```
                                                          23 26
Thermosynechococcus elongatus BP-1 MAPLRKTYVLKLYVAGNTPNSVRALKTLNNILEKEFKGVYALKVIDVLKNPQLAEEDKIL 60
Synechococcus sp. PCC 7942         MSP-RKTYILKLYVAGNTPNSVRALKTLKNILEVEFQGVYALKVIDVLKNPQLAEEDKIL 59
Cyanothece sp. PCC 7425            MSPLKKTYVLKLYVAGNTPNSVRALKTLNNILEQEFQGVYALKVIDVLKNPQLAEEDKIL 60
Microcystis aeruginosa             MSVFKKTYVLKLYVAGNTPNSVRALKTLKNILEEEFQGVYALKVIDVLKNPQLAEEDKIL 60
Synechocystis sp. PCC 6803         MSPFKKTYVLKLYVAGNTPNSVRALKMLKNILEQEFQGVYALKVIDVLKNPQLAEEDKIL 60
Trichodesmium erythraeum IMS101    MSPLKKTYVLKLYVAGNTPNSVRALKTLKEILEQEFQGVYALKVIDVLKNPQLAEEDKIL 60
Acaryochloris marina MBIC11017     MSSIRKTYVLKLYVAGNTPNSVRALRTLNHILETEFQGVYALKVIDVLKNPQLAEEDKIL 60
Cyanothece sp. PCC 8801            MVNFKKTYVLKLYVAGNTPNSVRALKTLKNILEDEFKGVYALKVIDVLKNPQLAEEDKIL 60
Cyanothece sp. PCC 7424            MNTFRKTYVLKLYVAGNTPNSVRALKTLKTILEQEFQGVYALKVIDVLKNPQLAEEDKIL 60
Synechococcus sp. WH 8102          -MSPRKTYILKLYVAGNTPNSMRALKTLRNILETEFRGVYALKVIDVLKNPQLAEEDKIL 59
Synechococcus sp. WH 7803          -MSPRKTYILKLYVAGNTPNSMRALKTLRNILETEFKGVYALKVIDVLKNPQLAEEDKIL 59
Synechococcus sp. PCC 7002         MNLLKKTYVLKLYVAGNTPNSVRALKTLKNILETDFKGVYALKVIDVLQNPQLAEEDKIL 60
Nostoc sp. PCC 7120                MNKARKTYVLKLYVAGNTPNSVRALKTLKNILEQEFQGIYALKVIDVLKNPQLAEEDKIL 60
Anabaena variabilis ATCC 29413     MNKARKTYVLKLYVAGNTPNSVRALKTLKNILEQEFQGIYALKVIDVLKNPQLAEEDKIL 60
Synechococcus sp. CC9311           -MSPRKTYILKLYVAGNTPNSMRALKTLRNILETEFKGVYALKVIDVLKNPQLAEEDKIL 59
                                      :***:***********:***: *. *** :*:*:*********:**********

                                                                 84     91
Thermosynechococcus elongatus BP-1 ATPTLAKVLPPPVRRIIGDLSNREKVLIGLDLLYEEIGDQAEDD-LGLE----------- 108
Synechococcus sp. PCC 7942         ATPTLAKVLPLPVRRIIGDLSDREKVLIGLDLLYGEL--QDSDDF--------------- 102
Cyanothece sp. PCC 7425            ATPTLAKILPPPVRKIIGDLSDREKVLIGLDLLYEEFAEAEAEAE--------------- 105
Microcystis aeruginosa             ATPTLAKVLPPPVRKIIGDLSDREKVLIGLDLLYEEIRERENDS---------------- 104
Synechocystis sp. PCC 6803         ATPTLAKILPPPVRKIIGDLSDREKVLIGLDLLYDEIREREAEDQ--------------- 105
Trichodesmium erythraeum IMS101    ATPTLSKILPPPVRKIIGDLSDREKVLIGLDLLYEELNEDEYNL---------------- 104
Acaryochloris marina MBIC11017     ATPTLAKVLPPPVRKIIGDLSDRERVLIGLDLLYEELSDGIMEY---------------- 104
Cyanothece sp. PCC 8801            ATPTLSKILPPPVRKIIGDLSDREKVLIGLDLLYEEIRERESEL---------------- 104
Cyanothece sp. PCC 7424            ATPTLAKVLPPPVRKIIGDLSDREKVLIGLDLLYDEIQDRESELDF-------------- 106
Synechococcus sp. WH 8102          ATPTLSKILPPPVRRIIGDLSDRERVLIGLDLLYDELADNAFSSG--------------- 104
Synechococcus sp. WH 7803          ATPTLSKILPPPVRRIIGDLSDRERVLIGLDLLYDELADNSLTSTLMDALEDADTTDSDS 119
Synechococcus sp. PCC 7002         ATPTLSKVLPPPVRKIIGDLSDREKVLIGLDLLYEEFLEREEEL---------------- 104
Nostoc sp. PCC 7120                ATPTLSKILPPPVRKIIGDLSDRERVLIGLDLLYEELTEEDWEAQSNL------------ 108
Anabaena variabilis ATCC 29413     ATPTLSKILPPPVRKIIGDLSDRERVLIGLDLLYEELTEEDWEAQSSL------------ 108
Synechococcus sp. CC9311           ATPTLSKILPPPVRRIIGDLSDRERVLIGLDLLYDELVDNDLNSSLMDALGVPDIEEADS 119
                                   *****:*:******:******:**:*********:*: :
```

Figure S1. Sequence alignment of KaiB from different species. Sequences were aligned using Clustal W. The residues involved in the KaiB–KaiB lateral interactions are highlighted in cyan (protomer 1) and pink (protomer 2).

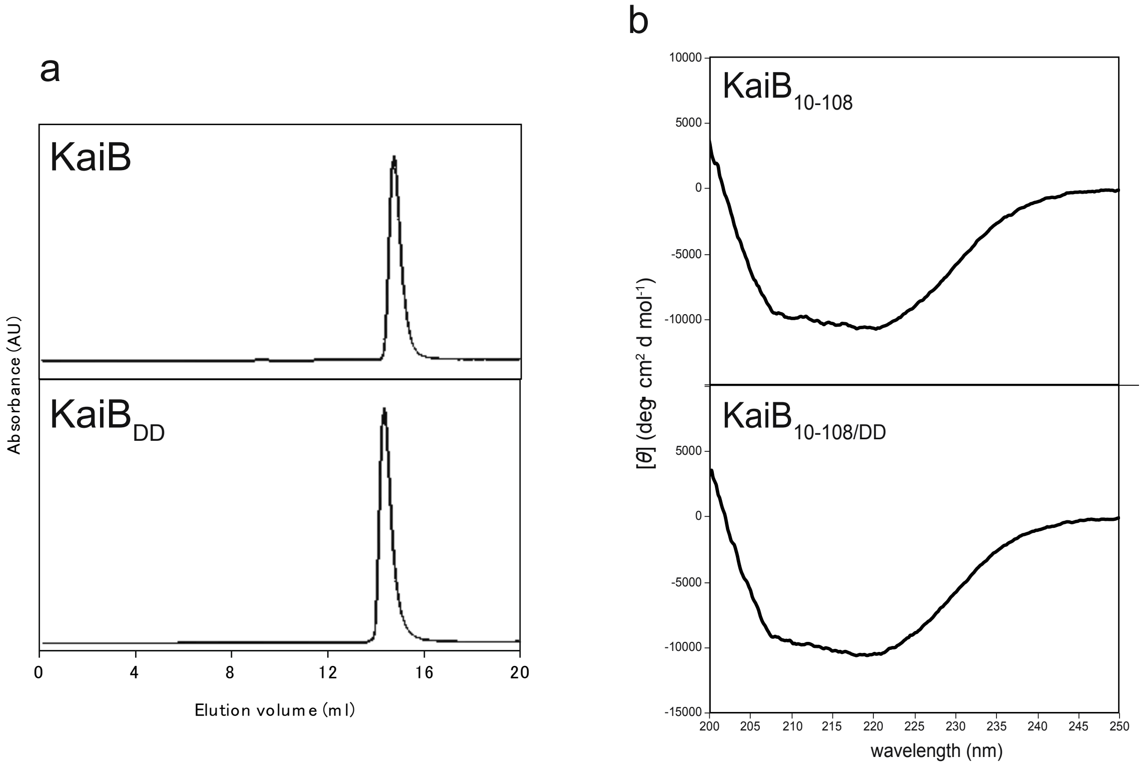

Figure S2. Comparative structural characterization between TeKaiB and TeKaiB$_{DD}$ by size exclusion chromatography and circular dichroism spectroscopy. (a) Elution profiles of TeKaiB and TeKaiB$_{DD}$. (b) Circular dichroism spectra of TeKaiB$_{10\text{-}108}$ and TeKaiB$_{10\text{-}108/DD}$.

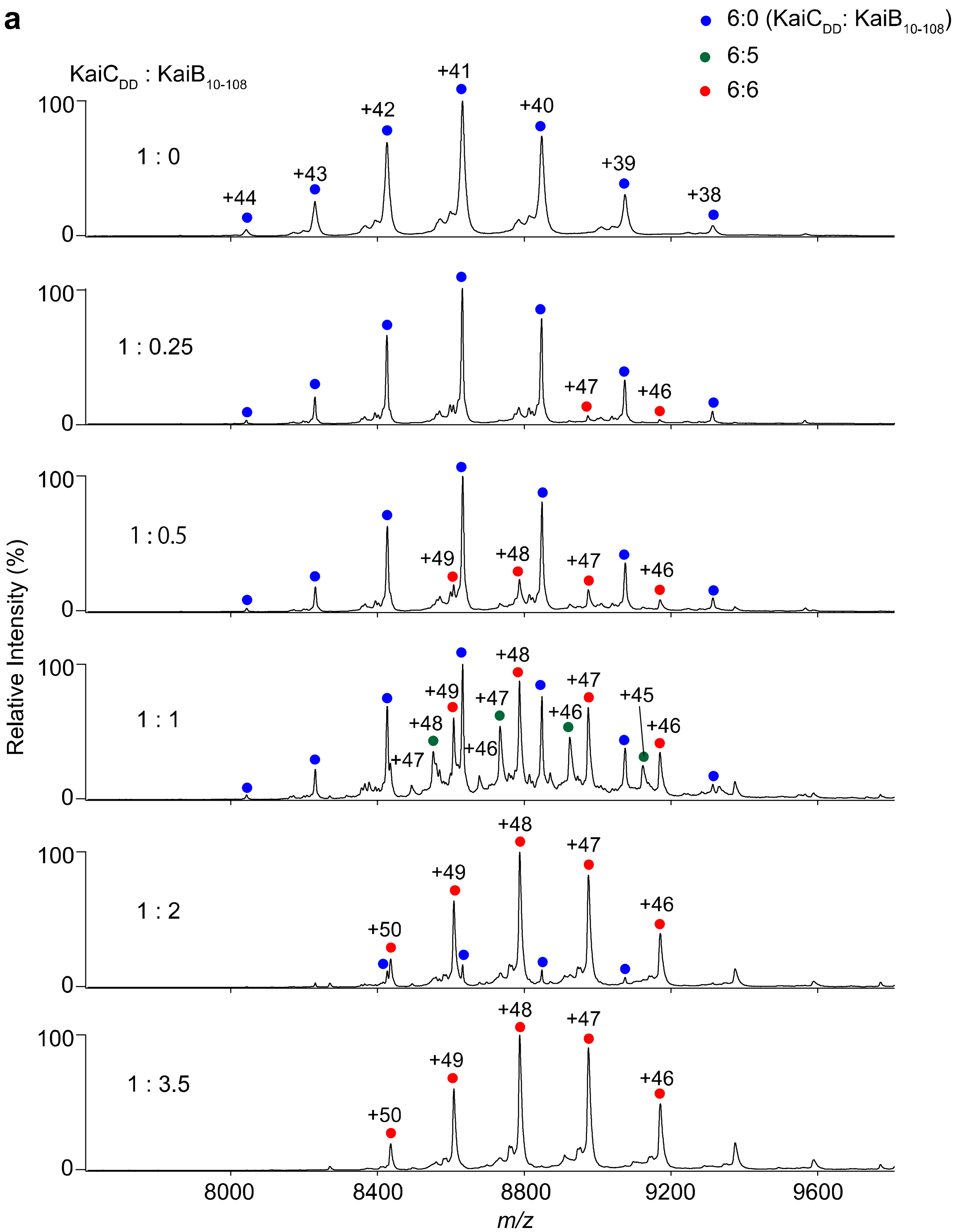
a
6:0 (KaiC$_{DD}$: KaiB$_{10-108}$)
6:5
6:6
KaiC$_{DD}$: KaiB$_{10-108}$
1 : 0
1 : 0.25
1 : 0.5
1 : 1
1 : 2
1 : 3.5
+44
+43
+42
+41
+40
+39
+38
+47
+46
+49
+48
+45
+50
Relative Intensity (%)
100
0
8000
8400
8800
9200
9600
m/z

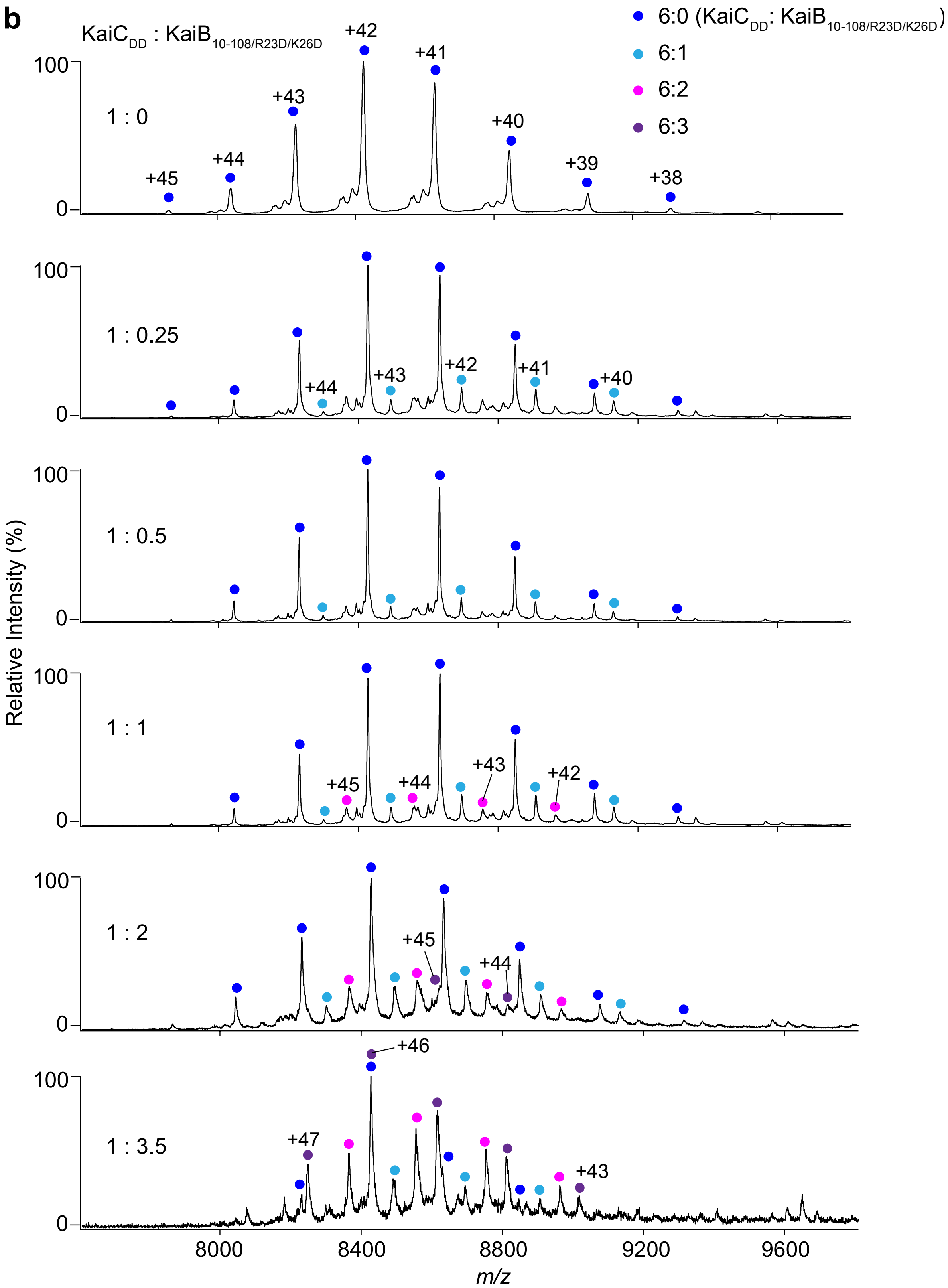

Figure S3. Native MS analysis of KaiB–KaiC complex formation. Mass spectra of mixtures of TeKaiC$_{DD}$ and (a) TeKaiB$_{10–108}$ or (b) TeKaiB$_{10–108/DD}$ at 1:0, 1:0.25, 1:0.5, 1:1, 1:2, and 1:3.5 molar ratios (TeKaiC$_{DD}$ to TeKaiB). The blue, cyan, magenta, purple, green and red circles show the ion series of the TeKaiC$_{DD}$ homohexamer and 6:1, 6:2, 6:3, 6:5 and 6:6 complexes of TeKaiC$_{DD}$ and the TeKaiB mutants, respectively.

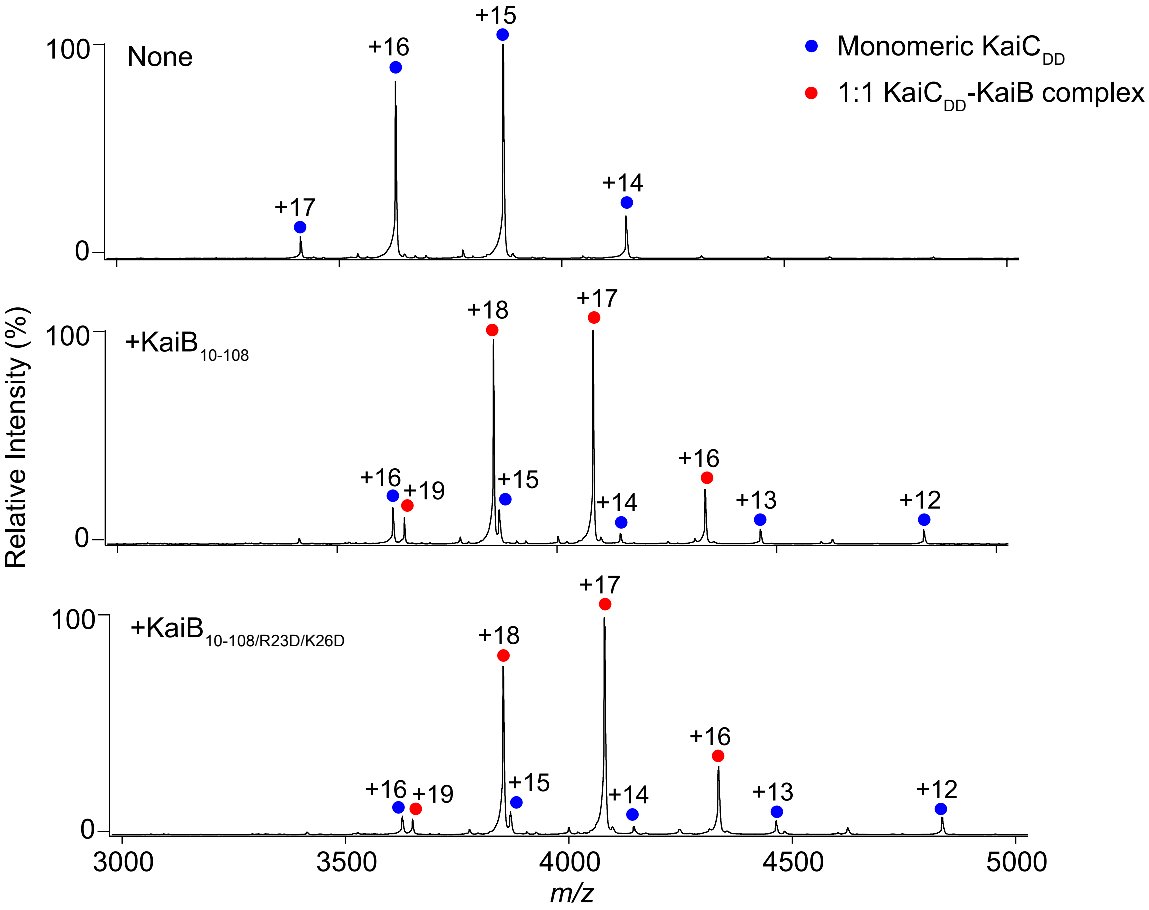

Figure S4. Native MS analysis of complex formation between the TeKaiB mutants and the TeKaiC$_{DD}$ monomer. KaiC$_{DD}$ monomer was monomeric in the absence of ATP conditions. Mass spectra of mixtures of the monomeric TeKaiC$_{DD}$ with TeKaiB$_{10–108}$ or TeKaiB$_{10–108/DD}$ at a 1:2 molar ratio (TeKaiC$_{DD}$ to TeKaiB). The blue and red circles show the ion series of the TeKaiC$_{DD}$ homo-hexamer and the 1:1 complex of TeKaiC$_{DD}$ and TeKaiB, respectively.

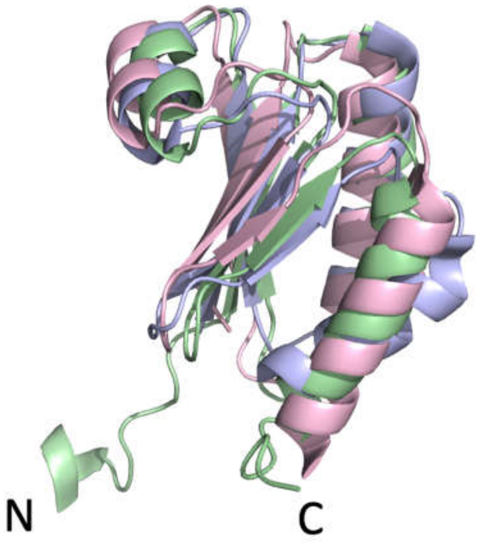

Figure S5. Structural alignment of the KaiB and SasA proteins. Superposition of SySasA$_N$ (PDB code: 1T4Y, shown in pale green), SyKaiB in the KaiC–KaiB complex (PDB code: 5N8Y, light pink), and TeKaiB in the KaiC–KaiB complex (PDB code: 5JWO, pale blue).

(a)

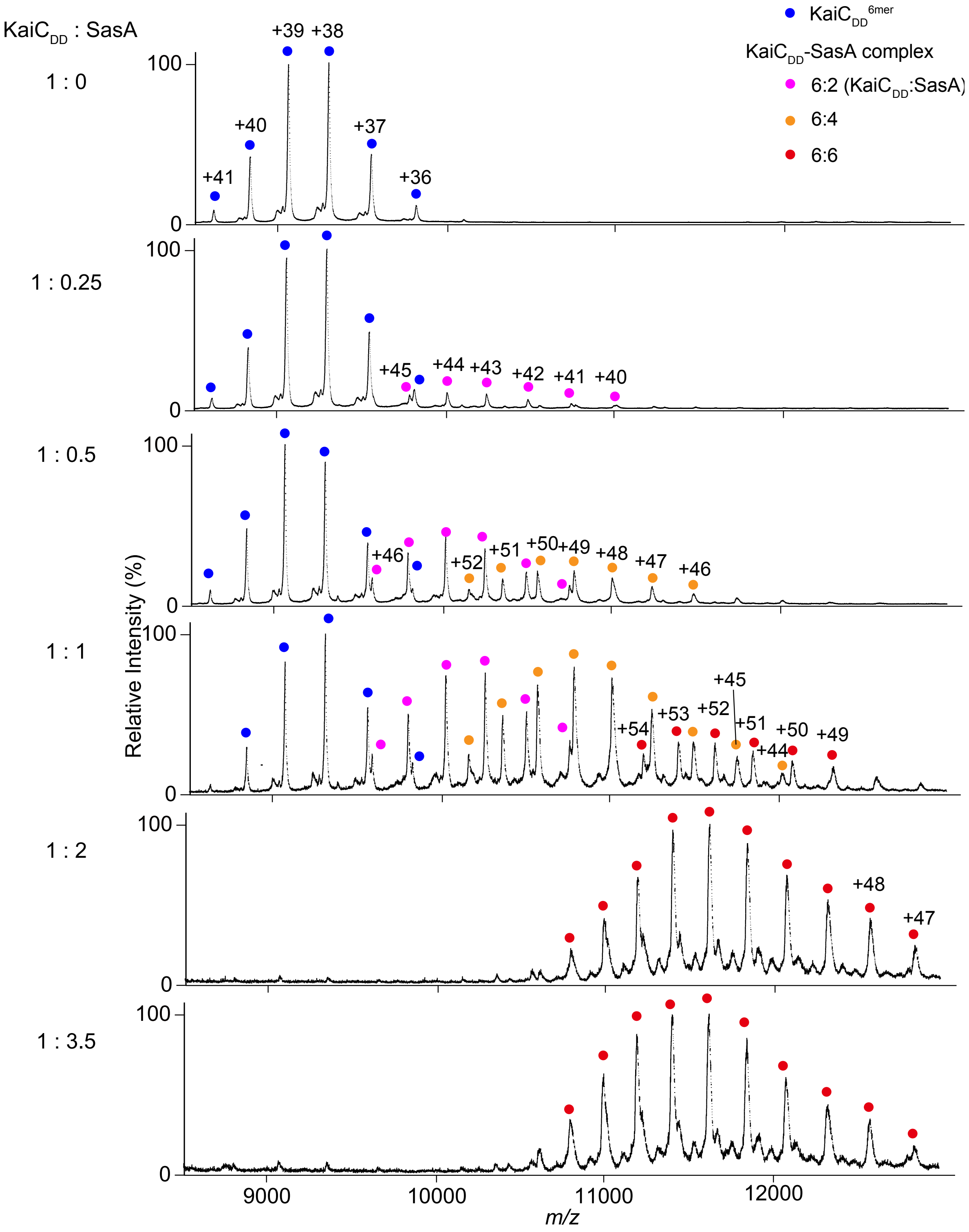
KaiC$_{DD}$: SasA
1 : 0
1 : 0.25
1 : 0.5
1 : 1
1 : 2
1 : 3.5
KaiC$_{DD}^{6mer}$
KaiC$_{DD}$-SasA complex
6:2 (KaiC$_{DD}$:SasA)
6:4
6:6
Relative Intensity (%)
m/z
9000
10000
11000
12000
+41 +40 +39 +38 +37 +36
+45 +44 +43 +42 +41 +40
+46 +52 +51 +50 +49 +48 +47 +46
+54 +53 +52 +45 +51 +44 +50 +49
+48 +47

(**b**)

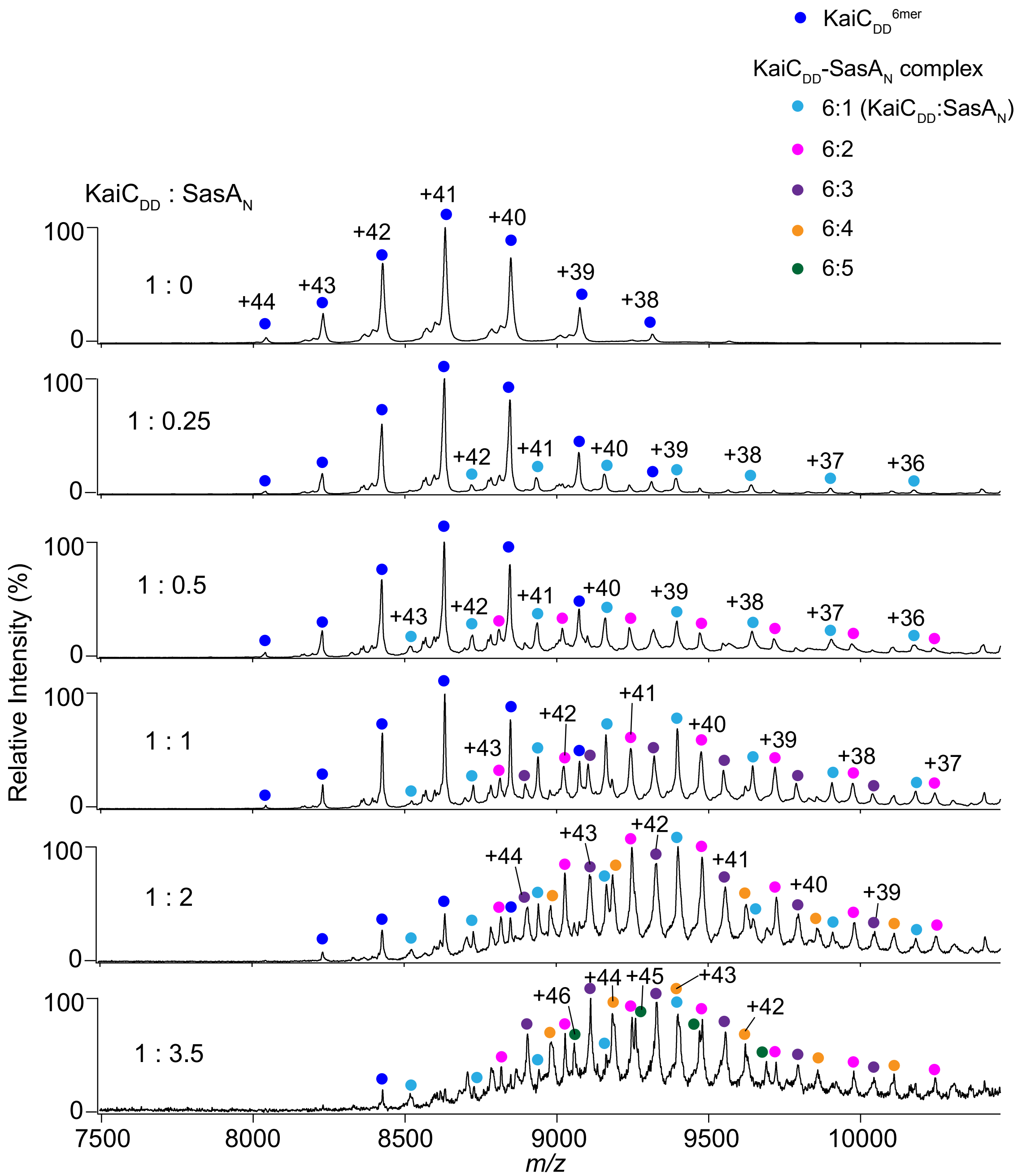

Figure S6. Native MS analysis of SasA–KaiC complex formation. Mass spectra of mixtures of TeKaiC$_{DD}$ with (a) full length TeSasA and (b) TeSasA$_N$ at 1:0, 1:0.25, 1:0.5, 1:1, 1:2, and 1:3.5 molar ratios (TeKaiC$_{DD}$ to TeSasA). The blue, cyan, magenta, purple, orange, and green circles show the ion series of the TeKaiC$_{DD}$ homo-hexamer and the 6:1, 6:2, 6:3, 6:4 and 6:5 complexes of TeKaiC$_{DD}$ and TeSasA, respectively.

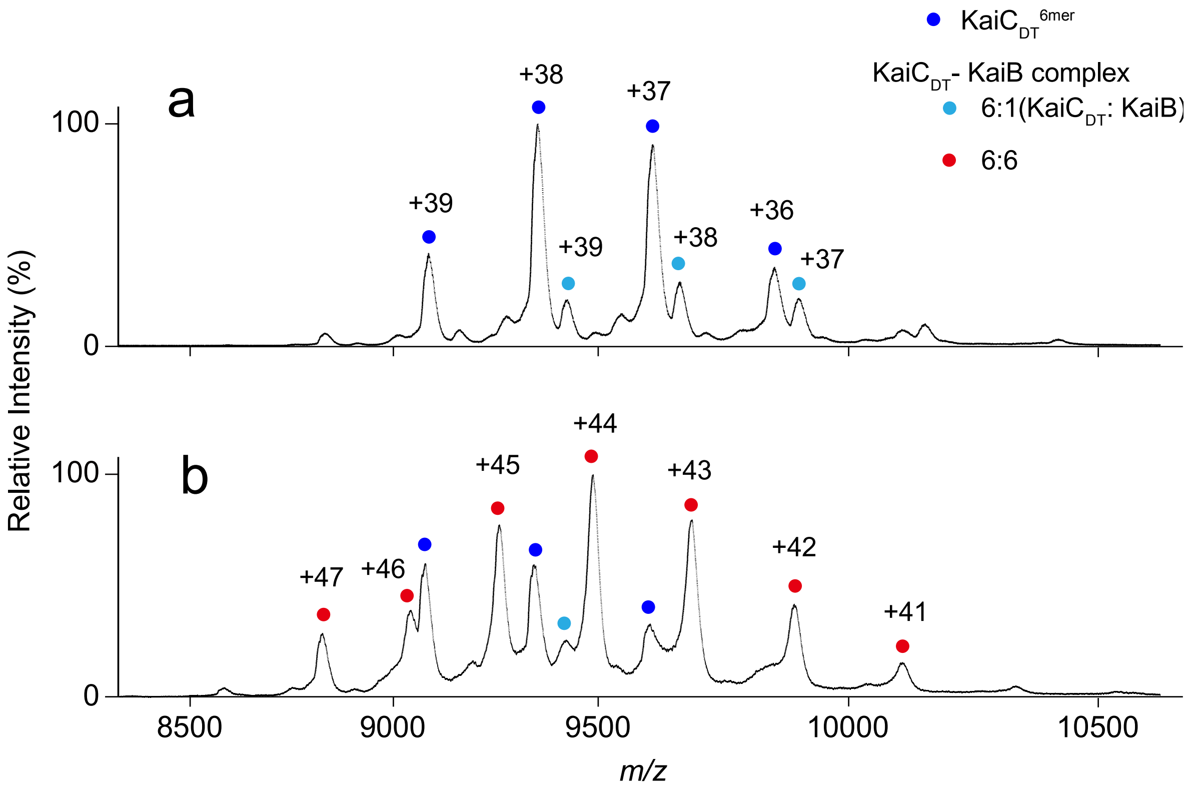

Figure S7. Native MS analysis of synechococcal KaiB–KaiC complex formation. Mass spectra of mixtures of SyKaiCDT with (a) SyKaiBDD or (b) SyKaiB at 2:3 molar ratio (SyKaiCDT to SyKaiB). The blue, cyan, and red circles show the ion series of the TeKaiCDD homohexamer and 6:1 and 6:6 complexes of SyKaiCDT and the SyKaiB mutants, respectively.

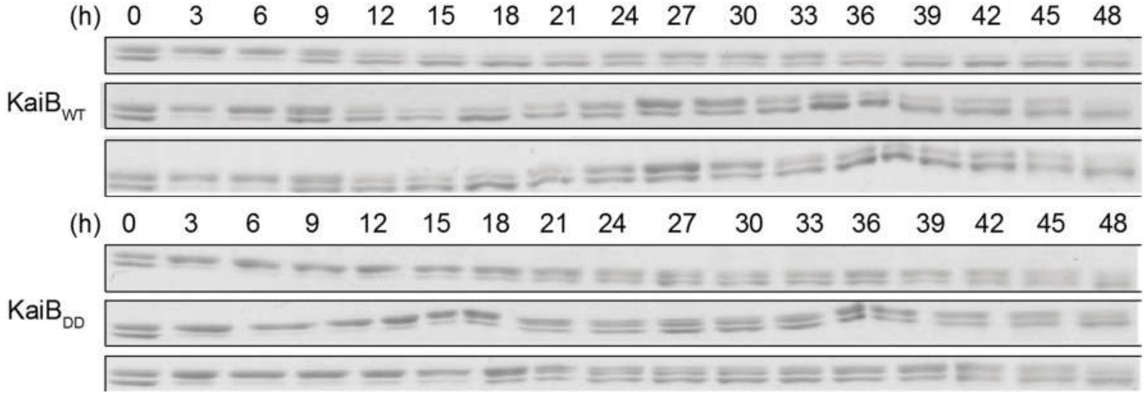

Figure S8. Circadian oscillations *in vitro*. Phosphorylated KaiC levels in the presence of wild-type SyKaiB and its mutant SyKaiBDD. SDS-PAGE of reaction mixtures containing SyKaiA, SyKaiC, wild-type KaiB, or KaiB mutant on 7.5% gels stained with Coomassie brilliant blue. The mixtures were incubated at 30°C for 48 h; aliquots were removed every 3 h and the reactions were quenched. The upper and lower bands correspond to phosphorylated and unphosphorylated KaiC, respectively. The data were presented in triplicate.
